# Ionization Energies of Polycyclic Aromatic Hydrocarbons: Benchmarking Semiempirical, Tight Binding, and Composite Methods

**DOI:** 10.1002/cphc.70622

**Published:** 2026-11-12

**Authors:** Alexandre Giuliani, Helgi Rafn Hrodmarsson

**Affiliations:** ^1^ Synchrotron SOLEIL Gif‐sur‐Yvette France; ^2^ Transform Department, UAR1008 INRAE Nantes France; ^3^ CNRS Université Paris Est Creteil and Université Paris Cité Créteil France

**Keywords:** composite methods, DFTB, ionization energy, polycyclic aromatic hydrocarbons, semi empirical

## Abstract

We benchmark semiempirical (PM6, PM7, RM1), tight‐binding (DFTB2, DFTB3, xTB‐IPEA, g‐xTB), and composite methods (HF‐3C, B97‐3C, PBEH‐3C, B3LYP‐3C, R2SCAN‐3C, *ω*B97X‐3C) for first, second, and double ionization energies of selected polycyclic aromatic hydrocarbons (PAHs). All calculations use the ΔSCF approach with zero‐point energy corrections. No single method is optimal across all ionization types. For first ionization energies, xTB‐IPEA is the most accurate approximate method, while *ω*B97X‐3C outperforms all composite approaches. *ω*B97X‐3C and DFTB3 show the most even performance across the three ionization categories. Comparison of B3LYP‐3C with literature B3LYP results reveals that composite corrections negligibly affect ionization energies, which are dominated by delocalization error effectively mitigated by range‐separated functionals. These results guide the selection of cost‐effective methods for PAH ionization studies.

## Graphical abstract

How do affordable quantum chemical methods perform for ionizing PAHs once, twice, or all the way to dications? Our benchmark of 13 methods reveals method dependences on charge state. Overall, range‐separated ωB97X‐3c and tight‐binding DFTB3 emerge as the most balanced choices for PAH ionization energy modeling.

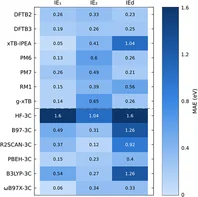

## 1 Introduction

Polycyclic aromatic hydrocarbons (PAHs) are recognized as major molecular constituents of the interstellar medium. They have been proposed as the carriers of the so‐called unidentified infrared bands observed in a wide range of astrophysical environments, including reflection nebulae, planetary nebulae, and photodissociation regions [1, 2, 3, 4, 5, 6]. Individual PAHs have also been identified in cold molecular clouds via their microwave spectra [7, 8, 9, 10, 11, 12, 13, 14, 15, 16, 17]. PAHs are considered fundamental building blocks at the interface between interstellar gas and dust [18, 19, 20], playing a central role in the energy balance, chemistry, and charge‐state distribution of the interstellar medium [21].

In these environments, PAHs are exposed to intense ultraviolet radiation fields, leading to photoionization of PAHs rather than fragmentation [22]. Consequently, PAHs control the ionization balance in astrophysical conditions. Their ionization states also strongly influence their infrared emission spectra, chemical reactivity, and interaction with surrounding matter. PAH clusters are also believed to play an active role as small carbonaceous grains [23]. The photoionization of PAH clusters has thus also been studied experimentally and theoretically [24, 25]. They are suggested to play an important role in the formation of prebiotic molecules as they provide shielding from ionizing radiation on the dust grains on top of which they form [26]. Accurate ionization energies are therefore essential for modeling PAH evolution, interpreting astronomical observations, and understanding their role in interstellar chemistry.

Considerable experimental and theoretical effort has been devoted to the determination of the first ionization energy of PAHs. Numerous studies [27, 28, 29, 30, 31, 32, 33, 34] have reported first vertical and adiabatic ionization energies, and several databases now provide reference values of benchmark quality for a wide range of PAH molecules [28]. Quantum chemical methods may consequently be validated against these datasets, which have contributed much to the present understanding of the ionization of PAHs. Second and double ionization energies, in contrast, have received far less attention, although they are available in the online theoretical spectral database of PAHs [35]. Their experimental determination is difficult, and, as a result, the available data remain sparse.

Second and double ionization energies are nevertheless of particular importance in astrophysical contexts. Under strong radiation fields, multiple charged PAHs may be formed and may significantly affect the charge balance and the survival of the molecules [36]. Experimental studies using ion action spectroscopy and charge‐stripping techniques have reported the second and, in some cases, the third ionization energies for a limited number of PAHs, namely, anthracene, pyrene, tetracene, coronene, ovalene, and dicoronylene [37, 38, 39, 40, 41]. Complementary theoretical investigations based on density functional theory have provided reference values for selected systems, but comprehensive datasets are still lacking [27, 28, 29]. It appears from those studies that the higher‐order ionization energies are sensitive to the molecular size and the electronic structure. Accurate and efficient computational approaches are therefore needed, particularly as one moves towards even larger PAHs such as those recently imaged in the Ryugu asteroid [42].

The question then arises as to how such energies can be most efficiently computed for the PAHs of astrophysical interest. High‐level ab initio methods rapidly become prohibitive as the size and the complexity of the molecules grow. Hence, it is worth benchmarking approximate quantum chemical approaches, such as the semiempirical and tight‐binding methods, which are considerably cheaper while retaining a quantum‐mechanical description of the electronic structure [43]. Such a benchmark strategy is well established for other key observables of astrophysical PAHs, such as their infrared spectra, for which the use of DFT and reparametrized semiempirical methods has been evaluated in previous work [44, 45, 46].

Composite methods constitute another family of efficient alternatives, in which small basis sets are combined with carefully designed correction schemes [47]. They have been devised so as to balance accuracy against computational cost, and they may be applied nonexclusively to any large molecular system [48]. Their use is growing, and it is consequently timely to assess their reliability for ionization energy calculations, notably for open‐shell and multiply charged species.

In the present work, a selection of semiempirical, tight‐binding, and composite methods has been benchmarked against available experimental and high‐level theoretical data, for the first, second, and double ionization energies of the series of PAHs shown in (Figure 1). The next section is devoted to the theoretical methods and to the computational protocols. The benchmarking results are then presented and discussed.

**FIGURE 1:**
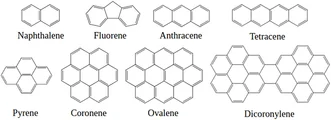
Chemical structures of the molecular species investigated.

## 2 Theoretical Methods

This work aims at assessing the performance of approximate and composite quantum chemical methods for the prediction of the first, second, and double ionization energies of selected PAHs. The set of molecular species has been chosen based on the availability of experimental data, especially with regard to second ionization energies. We also include high‐level theoretical reference values reported in the literature. All the ionization energies have been computed within the *Δ*SCF approach, which is well suited to charged and open‐shell species and which allows direct comparison with the experimental adiabatic ionization energies.

Zero‐point energy (ZPE) corrections were included whenever possible as they are known to systematically improve agreement with experimental data. An exception is the g‐xTB method, for which technical limitations prevent the calculation of vibrational frequencies and ZPE contributions.

### 2.1 Semiempirical and Tight‐Binding Methods

Several modern semiempirical and tight‐binding methods have been benchmarked, which are commonly used for large molecular systems on account of their computational efficiency [43, 49]. The PM6 [50], PM7 [51], and RM1 [52] semiempirical methods have been selected. Those approaches rest upon parameterized Hamiltonians derived from higher‐level theories and have been optimized so as to reproduce experimental thermochemical and electronic properties.

Tight‐binding methods were represented by DFTB2 [53] and DFTB3 [54] using the mio [53] and 3ob parameter sets [55], respectively. DFTB3 includes third‐order corrections to the Hamiltonian, enabling a more refined description of charge redistribution and electronic interactions, with the ThirdOrderFull option and derivatives of the Hubbard parameters (HubbardDerivs) of C = −0.1492 and H = −0.1857. We also tested the xTB Hamiltonian xTB‐IPEA [56], which incorporates an empirical correction optimized for vertical ionization energy calculations. The recently released g‐xTB method [57], which is still under development, has also been tested.

### 2.2 Composite Methods

Additionally, a set of composite methods has been evaluated [47, 58, 59, 60, 61, 62]. In those methods, relatively small basis sets are combined with dispersion, basis set superposition error, and short‐range corrections, so as to provide efficient alternatives to conventional DFT calculations.

These include HF‐3C [58], which uses the so‐called MINIX basis set [63] and to which three correction terms are added to the Hartree–Fock energy, namely, (a) the D3(BJ) dispersion correction [64, 65] to include London dispersion interactions, (b) a geometrical counterpoise (gCP) correction [66] to account for BSSE, and (c) a short‐range correction term to mitigate basis‐set deficiencies [58]. B97‐3C [60] is a revised version of the B97 GGA functional including the D3(BJ) dispersion correction [64, 65], a short‐range bond‐length correction [58], and a modified, reduced triple‐zeta basis set termed def2‐mTZVP [67]. PBEH‐3C [59] is a composite DFT method using a reparameterized PBE functional and a polarized double‐zeta basis set with D3 [64, 65] and gCP [66] corrections. B3LYP‐3C [61] is a variant of PBEH‐3C in which the PBEH functional is replaced by B3LYP, which improves the accuracy of vibrational frequencies. R2SCAN‐3C [62] is based on the meta‐GGA R2SCAN functional, complemented by the D4 and gCP corrections. It employs a newly developed basis set, mTZVPP, which is a variant of the mTZVP basis set from B97‐3C, itself derived from def2‐TZVP. *ω*B97X‐3C [47] is a composite range‐separated hybrid functional derived from the *ω*B97X‐V [68] functional. It incorporates D4 dispersion corrections [69, 70] and uses an optimized polarized valence double‐*ζ* basis set. *ω*B97X‐3C is the most sophisticated composite method so far.

### 2.3 Computational Details

Geometry optimizations, vibrational frequency calculations, ZPE corrections, and total energy evaluations were performed using ASE (v3.23) [71]. No scaling factor was applied to the vibrational energies. This ensures that the molecular geometries are converged using the same criterion, namely $f_{\max}=0.0005$, and that the vibrational energies are calculated with the same procedure across the different methods and are therefore comparable.

DFTB and xTB calculations were carried out with DFTB+ (v23.1) [72], while semiempirical calculations employed OpenMOPAC [73] (v23.0.1), the open‐source implementation of MOPAC [74]. The g‐xTB executable used was the preliminary g‐xTB(EEQ BC) version (August 12 2025) [57].

Composite‐method calculations were performed using ORCA (v6.1.0) [75, 76, 77, 78]. All the open‐shell species, that is, the doublet radical cations and the triplet dication of coronene, have been treated in the unrestricted scheme: UHF for HF‐3C and UKS for the five density functional‐based composite methods. This is the scheme for which those methods have been parameterized, and it is the one to which all the values reported hereafter refer. Restricted open‐shell Hartree–Fock (ROHF) single‐point energies have additionally been computed for the HF‐3C cations, by means of the %scf HFTyp ROHF end block. Those energies serve a diagnostic purpose only. Indeed, ORCA 6.1.0 does not provide analytical ROHF gradients so that a consistent set of ROHF geometries and harmonic frequencies could not be obtained for the largest systems at a reasonable computational cost. The difference between the ROHF and the UHF energies, taken at a common geometry, is reported in Table S5 and shall be used below to quantify the breaking of spin symmetry in the HF‐3C cations.

DFTB calculations used an electronic temperature of 300 K to improve self‐consistent charge convergence and ensure consistency with semiempirical reference states.

The electronic ground state of each dication, singlet or triplet, has been taken from the online theoretical spectral database of PAHs of Malloci et al. [35], which reports the spin multiplicities of the doubly ionized species over a wide set of PAHs. Within the present set, only the coronene dication has a triplet ground state. For dicationic species, triplet states were treated in DFTB + using colinear spin polarization, the atomic spin constants being set to −0.02265 for carbon and −0.07174 for hydrogen. In MOPAC, ORCA, and g‐xTB calculations, unrestricted formalisms were employed when required with the appropriate multiplicity.

A word may be said about the computational effort that those methods demand since it is the very reason for which the approximate ones are considered here. The cost of a full protocol is not a quantity fully proper to a method, as it depends on the starting geometry, on the convergence criteria, and on the machine. A single evaluation of the energy, on the other hand, may be compared from one method to the next. Such evaluations have been timed from scratch on a single core for coronene, ovalene, dicoronylene, and circumcircumcoronene (120 atoms) and are reported in Figure S1. For coronene, the semiempirical and tight‐binding methods lie within a factor of five of one another, whereas the composite methods span from two hundred to twenty‐seven thousand times DFTB2. Every method scales as an effective power of the number of atoms between about 1.8 and 2.7, so that this hierarchy is preserved up to 120 atoms, where the ratio between *ω*B97X‐3C and DFTB2 reaches $3.8\times 10^{4}$. Those figures are orders of magnitude and nothing more, but they place the accuracies discussed above in their proper context: the composite method, which performs best for the first ionization energy, is also the one that costs some four orders of magnitude more than the tight‐binding methods, which are themselves accurate to within one or two tenths of an eV.

The computational resources were accessed through the DARTS framework [79] provided by the GRADES group at SOLEIL.

## 3 Results and Discussion

The total energies, ZPE corrections, and vertical ionization energies for all species and methods are collected in Tables S1 and S2 of the Supporting Information. From these data, the adiabatic ionization energies with ZPE correction were derived using the ΔSCF approach (except for g‐xTB, for which no ZPE correction was applied; see above). Tables 1–3 present the resulting first, second, and double adiabatic ionization energies, respectively, alongside experimental and literature theoretical values. Figures 2a,b summarizes the deviations from experiment as box plots, where median deviations, interquartile ranges, and outliers provide insight into systematic biases and precision for the approximate methods and Figures 2d–f provides similar metrics for the composite methods. The corresponding statistical metrics are reported in Table S3. Before comparing the individual methods, the resolution of the statistical metrics should be discussed. Two independent contributions affect these metrics: the limited number of experimental references (seven, six, and six for the first, second, and double ionization energies, respectively) and the reported experimental uncertainties. Combining both contributions (see Supporting Information), we find that the smallest significant difference in mean absolute error (MAE) is about 0.1 eV for IE1, 0.15 eV for IE2, and 0.3 eV for IEd. Differences below these values are not used to rank the methods.

A central outcome of this benchmarking study is that no single method performs optimally for all three ionization energies. Instead, performance strongly depends on the ionization level and the underlying approximations of each method.

### 3.1 First Ionization Energies

The first adiabatic ionization energy calculations with ZPE corrections are presented in Table 1 along with experimental data and previous theoretical works. The (R) CCSD(T)‐F12/aug‐cc‐pVDZ and M06‐2X/6‐311+G(d) values were reported by Bourgalais et al. [29] provide high‐level reference points. Over their full set of PAHs and substituted analogs, the authors reported an MAD of 0.04 eV and an MSD of 0.0005 eV for the (R) CCSD(T)‐F12 method, confirming its near‐benchmark accuracy and the reliability of the experimental reference data. For the four molecules common to both studies (naphthalene, fluorene, anthracene, and pyrene), the CCSD(T)‐F12 values deviate from experimental values by 0.02–0.04 eV, consistent with this general performance. However, such calculations are computationally prohibitive beyond pyrene‐sized systems and cannot be extended to the larger PAHs of astrophysical interest.

**TABLE 1:** Adiabatic first ionization energies (in eV) with zero‐point energy correction (except for g‐xTB) for the set of PAHs studied in this work. Literature experimental and theoretical values are indicated by their respective references. No data were available for dicoronylene. Unlabeled values correspond to the present calculations.

	Naphthalene	Fluorene	Anthracene	Pyrene	Tetracene	Coronene	Ovalene	Dicoronylene
Experimental [80]	8.144(1)	7.91(2)	7.439(6)	7.426(1)	6.97(5)	7.29(3)	6.71	
xTB‐GFN2 [30, 32, 33]	12.92		12.14	12.14	11.62	11.92	11.36	
xTB‐GFN2 corr [30, 32, 33].	8.83		7.87	7.92	7.22	7.61	6.795	
HF‐3C [81]					5.68			
B97‐3C [81]					6.37			
PBEH‐3C [81]					6.70			
BLYP 6‐31G(d) [82]	7.38		6.57		6.03	6.49		
M06‐2X/6‐311 + G(d) [29]	8.141	7.918	7.388	7.431		7.393	6.776	
B3LYP 6‐31 + G^*^ [28]	7.80	7.56	7.02	7.07	6.49	7.02	6.36	6.07
B3LYP 6‐31G(d) [82]	7.66		6.85		6.31	6.83		
B3LYP‐D3BJ/def2‐SVP [32]	7.734		6.945		6.412			
B3LYP 6‐31G(d,p) [81]					6.18			
B3LYP/6‐311G(d,p) [37]	7.82					6.98		
LC‐BLYP 6‐31G(d) [82]	7.95		7.14		6.59	7.22		
CAM‐B3LYP/aug‐cc‐pVDZ [32]	7.956		7.185		6.659			
CAM‐B3LYP‐D3BJ/aug‐cc‐pVDZ [30]				7.223		7.173	6.531	
(R)CCSD(T)F12/aug‐cc‐pVDZ [29]	8.121	7.881	7.400	7.396				
DFTB2	8.48	8.18	7.73	7.72	7.23	7.42	6.95	6.61
DFTB3	8.41	8.11	7.66	7.66	7.16	7.35	6.87	6.53
xTB‐IPEA corr.	8.12	7.95	7.36	7.39	6.86	7.24	6.72	6.45
PM6	8.15	7.89	7.60	7.57	7.20	7.39	6.95	6.47
PM7	8.28	8.09	7.69	7.66	7.29	7.58	7.09	6.86
RM1	7.88	7.72	7.30	7.27	6.81	7.20	6.63	6.13
g‐xTB	7.96	7.91	7.22	7.23	6.71	7.25	6.61	6.33
HF‐3C	6.79	6.31	5.97	5.82	5.35	5.87	4.61	1.21
B97‐3C	7.68	7.44	6.91	6.95	6.39	6.82	6.27	5.96
R2SCAN‐3C	7.80	7.55	7.02	7.06	6.50	6.97	6.40	6.11
PBEH‐3C	8.00	7.78	7.24	7.28	6.71	7.23	6.62	6.39
B3LYP‐3C	7.63	7.39	6.86	6.91	6.35	6.79	6.21	5.93
*ω*B97X‐3C	8.16	7.94	7.41	7.45	6.90	7.43	6.81	6.64

It is worth discussing the nature of the experimental reference values themselves, all of which are the evaluated ionization energies of the NIST Chemistry WebBook [80], the same source as that used by Bourgalais et al. [29]. It happens that the determinations behind those values are not of the same kind over the whole series. For naphthalene, anthracene, and pyrene, the evaluated values rest on threshold photoelectron and laser spectroscopy measurements resolved to the meV, which yield genuinely adiabatic ionization energies. In contrast, for tetracene, coronene, and ovalene, the evaluated values coincide with photoelectron determinations that the NIST compilation labels as vertical. The distinction follows the size of the molecule, which is not surprising as no resolved measurement has been reported beyond pyrene. Comparing an adiabatic calculation with a vertical measurement introduces a systematic bias, which may be estimated from the theoretical adiabatic and vertical values of Malloci et al. [28] to be 0.05–0.06 eV for those three molecules. Correcting the three references by that amount would shift the mean signed error of every method by +0.024 eV without altering the ordering of the methods. This is of the same order as the differences between the best‐performing methods and should be kept in mind when those are compared. The vertical ionization energies computed here for all methods are collected in Table S2 so that the reader may perform the conversion. We have nonetheless retained the NIST‐evaluated values as they stand since they constitute the reference set of the field.

The raw xTB‐GFN2 from the COMPAS project [30, 32, 33] clearly show the necessity of applying an empirical corrections to the results of some methods, which present deviations of ≈4.5 eV. After correction, the present IPEA1 results show better agreement with experimental data than literature‐corrected xTB‐GFN2 and the best agreement of all approximate methods, as seen in (Figure 2a). Several other semiempirical and tight‐binding methods—most notably RM1 and g‐xTB—exhibit small median deviations, indicating good agreement with reference values. PM6, PM7, and the DFTB methods tend to slightly overestimate the first ionization energy. However, PM6 and PM7 present the widest error distribution and, for PM7, the worst mean bias, making them the least suitable methods for such calculations of PAHs.

**FIGURE 2:**
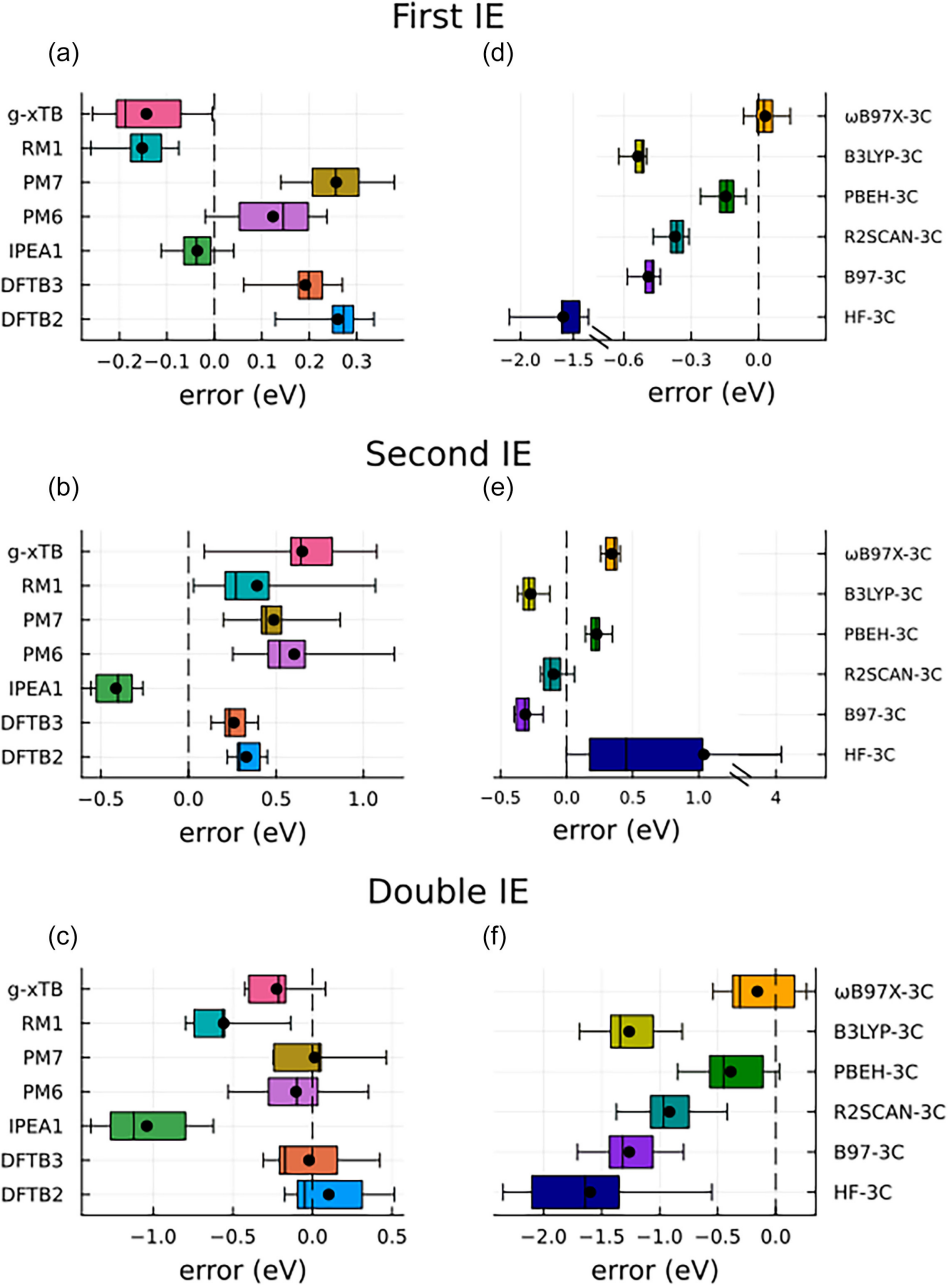
Boxplots of the errors (in eV) to the experimental data for semi empirical and tight binding methods for (a) the first, (b) second and (c) double ionization energies and for composite methods for (d) the first, (e) second, and (f) double ionization energies. The whiskers present the minimum and maximum deviation as range. The box shows the first and third quartile, the interquartile range thus containing 50% of the dataset. The black dot indicates the mean deviation and the vertical bar in the box shows the median deviation. The errors have been computed from the adiabatic ionization energies corrected for the zero‐point energy, consistently with Tables 1–3 and with Table S3, the only exception being g‐xTB, for which no zero‐point correction has been applied. In panels (d) and (e), the axis of errors is broken, as indicated by the double slashes. In panel (d), HF‐3C is separated from the five density functional composite methods by about 0.8 eV of empty range and the break restores a readable scale for the latter, while preserving the actual position of the former. In panel (e), the break accommodates the deviation of +4.0 eV obtained with HF‐3C for dicoronylene, which lies far beyond the range spanned by the other methods.

The ZPEs themselves deserve a word, as they are collected in Table S4 for the neutral species together with the correction ΔZPE = ZPE(cation) − ZPE(neutral) that enters the adiabatic ionization energy. Two facts emerge from the table. First, HF‐3C overestimates the ZPE of the neutral by 13% with respect to the mean of the five density functional composite methods and does so remarkably uniformly, from 12.9% for the ovalene to 13.7% for the fluorene. This is the well‐known overestimation of harmonic frequencies by Hartree–Fock, which is usually absorbed by an empirical scaling factor [84, 85]. The factors recommended for zero‐point vibrational energies lie between 0.914 and 0.925 at the Hartree–Fock level and close to 0.98 for the hybrid functionals [85], which would place the two families some 7% apart. None has been applied here since the quantity that enters the ionization energy is a difference of ZPEs, in which the overestimation cancels to a large extent. Moreover, the magnitude of *Δ*ZPE separates the two families of methods. It remains within ±0.08 eV for the five composite functionals, with mean values below 0.02 eV, whereas it reaches −0.12 to −0.15 eV on average for PM6, PM7, and RM1. As this correction enters the ionization energy directly, its weight is unequal from one family to the other, and it falls into three tiers rather than two. Were it omitted, the mean signed error of the first ionization energy would move by 0.11–0.14 eV for PM6, PM7, and RM1, by 0.04–0.06 eV for the tight‐binding methods, and by 0.02 eV at most for the five density functional composite methods. The zero‐point correction is therefore indispensable with the semiempirical methods, useful but modest with the tight‐binding ones, and may be neglected with the composite ones.

A notable characteristic of the DFTB methods is the narrow bias distribution compared to that of the other approximate methods across the series. Darghouth et al. [49] evaluated the performance of the DFTB3 method for first vertical ionization energy calculations for small‐ and medium‐sized molecules. In this study, the accuracy is obtained through an estimation of the goodness of a linear fit of the theoretical to reference data, which does not compare directly to the statistical metric used here, such as our RMSE of 0.20 eV for DFTB3 with ZPE correction (see Table S3). The authors reported an accuracy of ±0.489 eV (after linear calibration) for their large‐molecule set using SCC‐DFTB3 with 3ob parameters using the ΔSCF approach. Additionally, DFTB3 calculations on pentacene show a better agreement with experimental data than B3LYP/6‐31G^*^ calculations. This is in agreement with the results presented here. Overall, Darghouth et al. found that SCC‐DFTB3 is accurate enough for trends in large organic molecules, which is also supported by our findings.

DFT errors in the first ionization potentials for alkanes, alkenes, polyacenes, and PAHs have also been reported [82]. A systematic increase in error with molecular size was found and attributed to delocalization effects. For GGA functionals, errors as large as 1 eV were observed for oligoacenes. For PAHs, mean signed errors (MSEs) were −0.98 eV for BLYP, −0.61 eV for B3LYP, and −0.14 eV for LC‐BLYP. The B97‐3C and R2SCAN‐3C methods used here compare favorably to the GGA with lower MSE of −0.49 and −0.37, respectively (Figure 2d).

The present set allows for this size dependence to be examined more closely. Indeed, it contains two structural families that cover comparable ranges of size: the linear acenes, that is, naphthalene, anthracene, and tetracene, and the pericondensed PAHs, that is, pyrene, coronene, and ovalene. Table S6 separates them. The five density functional‐based composite methods drift by −0.08 to −0.12 eV along the acene series, from two to four fused rings, in agreement with the trend reported by Whittleton et al. [82] Along the pericondensed series, however, which spans a much wider range of carbon numbers, from *N*_C_ = 16 to *N*_C_ = 32 (where *N*_C_ stands for the number of carbon atoms), the deviation remains constant to within +0.02 to +0.07 eV. It appears, therefore, that the growth of the delocalization error follows the length of the linear conjugation rather than the number of carbon atoms. This is not unexpected as the HOMO–LUMO gap closes along the acene series, whereas compact PAHs retain a large gap. This distinction may be of consequence for astrophysical applications [86]. For the pericondensed family, the composite methods may be considered transferable in size, at least up to *N*_C_ = 32.

In our work, the MSE of B3LYP‐3C shows a slightly better value than the previous B3LYP calculations [82], and PBEH‐3C shows an improved MSE of −0.15 (Figure 2d). *ω*B97X‐3C also outperforms LC‐BLYP with an MSE value of 0.03, which is the best performance of all the methods tested here. HF‐3C, B97‐3C, and PBEH‐3c have been reported with MAE of 34.73 kcal/mol (1.5 eV), 3.75 kcal/mol (0.16 eV), and 7.84 kcal/mol (0.34 eV) [60] for the G21IP [87] set (36 adiabatic ionization energies of atoms and small molecule) of the GMNTKN55 [88] database. These results are consistent with the present ones (Table S3), for which HF‐3C, B97‐3C, and PBEH‐3C present MAE of 1.60, 0.49, and 0.15 eV, respectively. The agreement is close for the three methods, including the large error of HF‐3C, and the only difference is that we find PBEH‐3C to perform better than B97‐3C.

Beside the mean errors, Table S3 also reports *σ*, the standard deviation of the errors, that is, the scatter which remains once the systematic offset has been removed. Two distinct qualities of a method are thereby separated: how close it stands on average and how uniformly it behaves along the series. For the first ionization energy, the two families are comparable, *σ* lying between 0.05 and 0.10 eV for every method but HF‐3C, for which it reaches 0.25 eV. It happens that the composite functionals combine the smallest scatter of the whole set, 0.05 eV for B3LYP‐3C and B97‐3C, with the largest systematic offsets, −0.54 and −0.49 eV, respectively. Their ranking on the MAE alone consequently reflects the magnitude of their bias rather than their consistency, and a method such as B3LYP‐3C reproduces the variation of the ionization energy along the series more faithfully than its MAE would suggest. It should be stressed that no empirical shift has been applied to any of the methods discussed here. The only empirical correction used in this work is the one prescribed by the authors of the xTB‐IPEA method [56], without which the errors of that method would be considerably larger.

The relative standing of the two families depends on the ionization that is considered. For the first ionization energy, they are comparable. For the second one, the five density functional based composite methods are markedly more consistent, with σ ranging from 0.06 to 0.10 eV, than the semiempirical and tight‐binding ones, which range from 0.09 to 0.37 eV. HF‐3C is excluded from this comparison: with *σ* = 1.52 eV, it is by far the least transferable method of the whole benchmark for this property, for the reasons discussed below. This is not unexpected from parameterizations established on neutral molecules. For the double ionization energy, the ordering is reversed: g‐xTB and RM1 exhibit the smallest scatter, 0.19 and 0.27 eV, whereas every composite method lies between 0.35 and 0.68 eV. Hence, no method is at the same time unbiased and consistent for this last property.

In a comparison of several composite methods against tetracene [81], it has been reported adiabatic IE obtained by the ΔSCF method of 5.68, 6.70, 6.37, and 6.18 eV for HF‐3C, PBEH‐3C, B97‐3C, and B3LYP, respectively. These compare well with the present values of 5.35, 6.71, 6.39, and 6.35 eV for HF‐3C, PBEH‐3C, B97‐3C, and B3LYP‐3C. It is worth noting that the HF‐3C value of Praveen et al. lies 1.29 eV below the experimental ionization energy of tetracene, which is close to the deviation of −1.62 eV obtained here. Hence, the poor performance of HF‐3C for PAH radical cations is independently reproduced in the literature and may not be ascribed to an artifact of the present protocol. Among composite methods, *ω*B97X‐3C and PBEH‐3C provided the most balanced performance.

The behavior of HF‐3C deserves a closer look. Indeed, there is not a uniform loss of accuracy. HF‐3C underestimates the first ionization energy by −1.35 eV for naphthalene and by up to −2.10 eV for ovalene, and for dicoronylene, it returns an unphysical value of 1.21 eV, to be compared with 6.07 eV at the B3LYP/6‐31 + G^*^ level [28]. It happens that the error is confined to the open‐shell doublet cation. The unrestricted Hartree–Fock solution for the radical cation breaks the spin symmetry. This single defect propagates through the three observables in a way that is diagnostic. The cation being the final state of the first ionization and the initial state of the second one, the two errors are of equal magnitude and of opposite sign. The double ionization energy, which connects the neutral directly to the dication and never involves the cation, is left unaffected.

Two observations indicate that this is a property of the method rather than of the present protocol. First, the five density functional‐based composite methods have been run with the same unrestricted formalism, the same criteria of geometry optimization, and the same program, and none of them exhibits such a behavior. Moreover, *ω*B97X‐3C, which carries full Hartree–Fock exchange in the long range, is in fact the most accurate method of the whole benchmark. The breakdown may therefore be ascribed to the absence of correlation in the Hartree–Fock reference rather than to Hartree–Fock exchange as such. This is in line with the known tendency of Hartree–Fock to overestimate the polyradical character of extended π systems. Second, the symmetry breaking may be measured directly. The expectation value $\left\langle {\hat{S}}^{2}\right\rangle$ of the unrestricted solution, which should amount to 0.75 for a doublet, rises from 0.877 for the naphthalene cation up to 6.859 for that of dicoronylene, whose unrestricted wavefunction has consequently nothing left of a doublet. All these values are collected in Table S5. The energy gained by the broken solution, taken as the difference between the restricted open‐shell and the unrestricted Hartree–Fock energies at the same geometry, follows this contamination very closely. Both follow the compactness of the PAH rather than its size alone: the contamination remains between 0.13 and 0.21 for the acenes, whereas it reaches 0.60 for pyrene, 1.13 for coronene, 1.72 for ovalene, and 6.11 for dicoronylene. It is worth noting, finally, that the triplet dication of coronene is almost free of contamination, $\left\langle {\hat{S}}^{2}\right\rangle$ = 2.05 for an ideal value of 2.00, and that the corresponding energy gain does not exceed 0.17 eV. The defect is thus confined to the doublet cation, as the behavior of the three ionization energies had already suggested.

A direct comparison of the present B3LYP‐3C results with B3LYP calculations from the literature allows us to isolate the effect of the composite correction terms on the computed ionization energies. The B3LYP‐D3BJ/def2‐SVP values from the COMPAS project [32] are particularly relevant as def2‐SVP is the parent basis of the modified double‐zeta basis used in B3LYP‐3C. For naphthalene, anthracene, and tetracene, B3LYP‐D3BJ/def2‐SVP yields values systematically higher than B3LYP‐3C by 0.07–0.11 eV, indicating that the basis set modification, gCP, and short‐range corrections in the composite scheme slightly lower the computed ionization energies relative to standard def2‐SVP. For comparison, the B3LYP/6‐31G(d) values were reported by Whittleton et al. [82] agree with B3LYP‐3C to within 0.01–0.04 eV. These use a basis set of comparable quality but include no dispersion or counterpoise corrections. Hence, the individual correction terms largely cancel for this property. In all cases, these differences remain small compared to the systematic underestimation of 0.5–0.6 eV relative to experiment, confirming that the composite corrections have a negligible impact on the calculated first adiabatic ionization energies.

This result is consistent with the nature of the corrections involved. The D3 dispersion term addresses long‐range noncovalent interactions, which largely cancel in the ΔSCF energy difference between neutral and cationic species of similar geometry. The gCP correction for basis set superposition error will also partly cancel out energies between the neutral and cation as both states employ the same basis set. Finally, the short‐range bond‐length correction primarily affects equilibrium geometries rather than relative electronic energies. Consequently, for ionization energy calculations on PAHs, B3LYP‐3C essentially reduces to standard B3LYP at the double‐zeta level. The reported systematic underestimation of ionization energies in extended *π*‐conjugated systems [82, 89, 90] remains uncorrected by the composite scheme.

The M06‐2X/6‐311 + G(d) values were reported by Bourgalais et al. [29], which in our (Table 1) extend to coronene and ovalene, achieve deviations comparable to those of *ω*B97X‐3C (within 0.01–0.14 eV of experiment). Over their full molecular set, Bourgalais et al. reported a MAD of 0.05 eV for M06‐2X, close to that of CCSD(T)‐F12 (0.04 eV) but with a larger RMSD of 0.19 eV compared to 0.02 eV, indicating greater variability across the series. Notably, both M06‐2X and *ω*B97X‐3C mitigate the delocalization error [89, 90] that affects standard GGA and hybrid functionals for extended *π*‐systems. However, they do this through different strategies. M06‐2X relies on a high proportion of exact exchange (54%), whereas *ω*B97X‐3C employs range separation combined with a compact double‐zeta composite basis. The fact that *ω*B97X‐3C reproduces the accuracy of M06‐2X/6‐311+G(d) with a substantially smaller basis set highlights the effectiveness of range separation in mitigating delocalization errors for ionization energy calculations. This is consistent with the observation above that the composite corrections themselves have a rather small impact on this property.

The CAM‐B3LYP/aug‐cc‐pVDZ values from the COMPAS project [30, 32] provide an additional point of comparison as CAM‐B3LYP is also a range‐separated hybrid functional and it systematically underestimates the first ionization energy by 0.12–0.31 eV both with and without D3BJ dispersion correction. The CAM‐B3LYP/aug‐cc‐pVDZ values without dispersion correction show a size‐dependent increase in error from −0.19 eV for naphthalene to −0.31 eV for tetracene. This indicates a residual delocalization error. Notably, these values are very close to the LC‐BLYP/6‐31G(d) results of Whittleton et al. [82] (e.g., 7.956 vs. 7.95 eV for naphthalene; 6.659 vs. 6.59 eV for tetracene), despite the substantially larger aug‐cc‐pVDZ basis set. This shows that for range‐separated functionals, the basis set effect is small for this property. The performance of CAM‐B3LYP thus appears intermediate between that of standard hybrid functionals such as B3LYP (underestimation of 0.5–0.6 eV) and the more accurate *ω*B97X‐3C (within 0.01–0.14 eV of experiment). This suggests that the specific parameterization of the range separation plays a significant role in succeeding in more accurate calculations of PAH ionization energies.

Similarly, the xTB‐IPEA method achieves deviations of comparable magnitude (0.02–0.08 eV for the same four molecules as the F12 calculations) at a fraction of the computational cost of any DFT calculation. These comparisons reinforce the central finding that approximate and composite methods provide reliable and practical alternatives for ionization energy calculations on large PAHs, where high‐level ab initio approaches become inaccessible.

In conclusion, the best results for adiabatic ionization energy calculations for our set of PAHs are obtained for approximate methods (RM1 and IPEA1) and for *ω*B97X‐3C but at much higher computational costs.

### 3.2 Second Ionization Energies

The results of the calculations for the second ionization energies are presented in (Table 2). Unlike the first ionization energies, no high‐level theoretical reference values (CCSD(T)‐F12 or M06‐2X) are available for this property, so the benchmarking relies entirely on experimental data. The experimental second ionization energies of anthracene, pyrene, tetracene, coronene, ovalene, and dicoronylene have all been obtained in the same way as the appearance energies of the dications formed by photoionization of mass‐selected cations stored in an ion trap and thermalized at room temperature [40, 41]. The ionization yield of a cation rising sharply at threshold, these appearance energies are close to adiabatic values. In contrast, the naphthalene value derives from charge‐stripping mass spectrometry, which yields values 1–2 eV above photoionization thresholds [83] with an uncertainty of 0.5 eV. No experimental value is available for fluorene. Both molecules have therefore been left out of the statistical metrics of the second ionization energy, which rest on the six remaining species. In the same way, the metrics of the double ionization energy rest on the six molecules for which an experimental value exists, ovalene and dicoronylene being excluded, and those of the first ionization energy on the seven species other than dicoronylene. The values listed in Tables 1–3 for the excluded species are reported for completeness but do not enter any average. The coronene dication ground state is a triplet state [37], which represents an additional difficulty.

**TABLE 2:** Adiabatic second ionization energies (in eV) with zero‐point energy correction (except for g‐xTB) for the set of PAH studied. Experimental and theoretical data from the literature are indicated by their respective references. No data were available for fluorene. Unlabeled values correspond to the present calculations.

	Naphthalene	Fluorene	Anthracene	Pyrene	Tetracene	Coronene	Ovalene	Dicoronylene
Experimental	14.6(5) [83]		11.7(1) [40]	11.7(1) [40]	10.9(1) [40]	10.95(5) [40]	10.0(1) [41]	9.1(2) [41]
B3LYP 6‐31+G^*^ [28]	13.19	12.39	11.68	11.57	10.66	10.72	9.82	8.84
B3LYP/6‐311G(d,p) [37]	13.22					10.79		
DFTB2	13.54	12.77	12.15	11.99	11.20	11.39	10.28	9.32
DFTB3	13.51	12.72	12.10	11.94	11.13	11.30	10.20	9.23
xTB‐IPEA corr.	12.58	11.93	11.25	11.14	10.35	10.64	9.65	8.84
PM6	13.25	12.58	12.14	11.95	11.43	11.46	10.71	10.28
PM7	13.27	12.66	12.11	11.90	11.35	11.38	10.56	9.97
RM1	13.06	12.53	11.91	11.73	11.23	11.16	10.50	10.17
g‐xTB	13.63	12.67	12.78	12.40	11.49	11.04	10.58	9.96
HF‐3C	13.36	13.05	11.88	11.88	10.90	12.08	10.73	13.12
B97‐3C	13.03	12.24	11.52	11.42	10.50	10.62	9.71	8.70
R2SCAN‐3C	13.29	12.49	11.76	11.66	10.73	10.78	9.92	8.90
PBEH‐3C	13.53	12.77	12.05	11.95	11.04	11.15	10.24	9.28
B3LYP‐3C	13.07	12.27	11.57	11.47	10.56	10.66	9.72	8.73
*ω*B97X‐3C	13.51	12.76	12.11	11.98	11.16	11.33	10.36	9.46

Second ionization energies present a greater challenge for approximate methods than first ionization energies. Most semiempirical approaches show a systematic positive bias, whereas xTB‐IPEA slightly underestimates values, likely due to its empirical correction being optimized primarily for first ionization energies. PM6 and PM7 again show large overestimations, particularly for the larger PAHs (deviations exceeding 1 eV for dicoronylene). The g‐xTB method produces a notable outlier for anthracene (12.78 eV vs. experimental 11.7 eV), a deviation of more than 1 eV that contrasts with its otherwise reasonable performance for the first ionization energy. As for the first ionization energies, DFTB2 and DFTB3 exhibit remarkably narrow error distributions, indicating stable and predictable performance. The DFTB3 method achieves an RMSE of 0.27 eV for the second ionization energy, confirming its consistent accuracy across ionization types.

Among composite methods, R2SCAN‐3C, PBEH‐3C, and B3LYP‐3C provide the best results for the second ionization energies, in line with their performance for the first ionization energies. HF‐3C, conversely, overestimates the second ionization energy all along the series by 1.13 eV for coronene and by 4.02 eV for dicoronylene. This is the mirror image of the underestimation of the first ionization energy discussed above. A comparison of the present B3LYP‐3C results with the B3LYP/6‐31 + G^*^ values of Malloci et al. [28] shows systematic differences of 0.06–0.12 eV, with B3LYP‐3C yielding slightly lower values across the series. This is in contrast to the first ionization energies, where the two methods agreed within 0.01–0.04 eV. The larger discrepancy for the second ionization energy likely reflects the role of diffuse functions present in the 6‐31+G^*^ basis set, which are absent in the compact double‐zeta basis of B3LYP‐3C. For the transition from the mono‐ to dication, diffuse functions may provide a more flexible description of the electron density in the multiply charged species. This is an effect that does not cancel as effectively in the ΔSCF energy difference as it does for the neutral‐to‐cation transition. The B3LYP/6‐311G(d,p) values were reported by Schröder et al. [37] for naphthalene (13.22 eV) and coronene (10.79 eV) fall between B3LYP/6‐31 + G^*^ and B3LYP‐3C, consistent with the intermediate quality of this triple‐zeta basis set. Despite these basis set‐dependent variations, all B3LYP‐based methods underestimate the experimental values by similar amounts, confirming that the delocalization error remains the dominant source of error for the second ionization energy as well.

### 3.3 Double Ionization Energies

Table 3 presents the results of the double ionization energies. Experimental data are more limited than for the first and second ionization energies: no values are available for ovalene or dicoronylene, restricting the benchmark set to six molecules. As for the second ionization energy, no high‐level theoretical reference values are available.

**TABLE 3:** Adiabatic double ionization energies (in eV) with zero‐point energy correction (except for g‐xTB) for the set of PAHs studied in this work. Experimental data are missing for ovalene and dicoronylene. The coronene dication ground state is a triplet state as reported by Schroeder et al. [37]. Literature values are indicated by their respective references. Unlabeled values correspond to the present calculations.

	Naphthalene	Fluorene	Anthracene	Pyrene	Tetracene	Coronene	Ovalene	Dicoronylene
Experimental	21.5(2) [83]	21.0(2) [83]	20.1(2) [39]	19.8(2) [83]	18.6(2) [83]	18.5(2) [37]		
B3LYP 6‐31+G^*^ [28]	20.99	19.96	18.70	18.63	17.15	17.74	16.18	14.91
B3LYP/6‐311G(d,p) [37]	21.04					17.77		
DFTB2	22.02	20.95	19.88	19.71	18.43	18.81	17.23	15.94
DFTB3	21.92	20.83	19.76	19.59	18.29	18.66	17.08	15.76
xTB‐IPEA corr.	20.70	19.88	18.61	18.53	17.21	17.88	16.37	15.29
PM6	21.40	20.47	19.74	19.52	18.63	18.85	17.66	16.74
PM7	21.55	20.75	19.81	19.56	18.64	18.97	17.65	16.82
RM1	20.95	20.26	19.21	19.00	18.04	18.36	17.14	16.30
g‐xTB	21.58	20.57	19.99	19.63	18.20	18.29	17.19	16.30
HF‐3C	20.15	19.36	17.85	17.70	16.25	17.95	15.33	14.34
B97‐3C	20.71	19.68	18.43	18.37	16.89	17.44	15.98	14.66
R2SCAN‐3C	21.08	20.03	18.78	18.72	17.23	17.75	16.32	15.01
PBEH‐3C	21.53	20.55	19.28	19.23	17.75	18.39	16.86	15.67
B3LYP‐3C	20.69	19.66	18.44	18.38	16.91	17.44	15.93	14.66
*ω*B97X‐3C	21.66	20.69	19.52	19.43	18.06	18.77	17.16	16.10

For double ionization energies, the delocalization error compounds as the energy difference now spans two successive ionization steps. A comparison of the present B3LYP‐3C results with the B3LYP/6‐31 + G^*^ values of Malloci et al. [28] reveals systematic differences of 0.24–0.30 eV across the entire series, with B3LYP‐3C yielding lower values in all cases. These differences are substantially larger than those observed for the first (0.01–0.04 eV) and second (0.06–0.12 eV) ionization energies. The B3LYP/6‐311G(d,p) values were reported by Schroeder et al. [37] for naphthalene (21.04 eV) and coronene (17.77 eV) are consistent with this trend, falling between B3LYP/6‐31 + G^*^ and B3LYP‐3C.

Among approximate methods, several semiempirical and tight‐binding methods, particularly PM6, PM7, DFTB2, and DFTB3, yield median deviations close to zero and comparatively small spreads for the double ionization energies. This contrasts with the composite methods, where many exhibit systematic underestimation. The DFTB3 method confirms its consistent accuracy across all ionization types, with an RMSE of 0.28 eV for the double ionization energy. PM7, DFTB2, and g‐xTB reach 0.27 eV, which makes these methods equivalent within the resolution of the benchmark. Among composite methods, *ω*B97X‐3C and PBEH‐3C give the smallest errors (MAE of 0.37 and 0.47 eV, respectively). As for HF‐3C, whose failure for the first and second ionization energies originates in the cation, it recovers here a behaviour comparable to that of B97‐3C and B3LYP‐3C. Indeed, the double ionization energy connects the neutral directly to the dication and does not involve the open‐shell cation.

The performance of the present methods can be compared with the DIPCS10 set (10 adiabatic double ionization energies of closed‐shell atoms and small molecules) from the GMTKN55 database [88]. For this set, HF‐3C, PBEH‐3C, and B97‐3C have been reported with MAE of 64.22 kcal/mol (2.78 eV), 4.15 kcal/mol (0.18 eV), and 7.91 kcal/mol (0.34 eV), respectively [60]. The present double ionization MAE of 1.71, 0.47, and 1.33 eV for HF‐3C, PBEH‐3C, and B97‐3C show larger errors for PAHs than for the DIPCS10 set, consistent with the expected increase of delocalization error with molecular size. PBEH‐3C again outperforms B97‐3C, in agreement with the trend observed for the first ionization energies. R2SCAN‐3C presented 4.76 kcal/mol (0.21 eV) MAE on the DIPCS10 set [62], which compares to the present result of 0.98 eV for the double ionization energy of PAHs, confirming that the errors increase significantly when moving from small molecules to extended *π*‐conjugated systems.

## 4 Conclusion

In this work, we benchmarked a broad range of semiempirical, tight‐binding, and composite methods for calculating first, second, and double ionization energies of PAHs. Our results demonstrate that method performance is strongly dependent on ionization order and that no single approach is universally optimal.

xTB‐IPEA and *ω*B97X‐3C give the best first ionization energies, with MAE of 0.05 and 0.06 eV, respectively. PM6, g‐xTB, RM1, and PBEH‐3C follow as a group, with MAE of 0.13–0.15 eV. DFTB3, for its part, is among the methods whose errors over the three ionization orders are the smallest and the most uniform, with RMSE values of 0.20, 0.27, and 0.28 eV for the first, second, and double ionization energies, respectively. For the double ionization energy, it is indistinguishable from PM7, DFTB2, and g‐xTB. It should be kept in mind, however, that these metrics rest on seven, six, and six molecules only. Taking into account both the finite size of the reference set and the experimental uncertainties (up to ±0.2 eV), differences in MAE below 0.1, 0.15, and 0.3 eV for the first, second, and double ionization energies, respectively, are not significant and are not used to rank the methods. Among composite methods, *ω*B97X‐3C achieves the best accuracy for the first ionization energy (MSE of 0.03 eV). However, composite methods do not systematically outperform faster approaches for the second and double ionization energies, where DFTB3 remains competitive. HF‐3C should not be recommended for the open‐shell cations of large PAHs. The unrestricted solution for the radical cation breaks the spin symmetry, and the resulting error grows with the size of the molecule, up to 4.78 eV for dicoronylene. Its description of the closed‐shell dications, by contrast, remains comparable to that of the other composite methods.

Two practical consequences follow as regards the zero‐point correction. It should not be omitted when a semiempirical method is employed since ΔZPE amounts there to one or two tenths of an eV and shifts the computed ionization energy accordingly. It is smaller with the tight‐binding methods, around 0.04 eV on average, and for the composite ones, it stays on average below 0.02 eV, so that in both cases it may be dispensed with. This is of some practical weight, as it spares the calculation of the harmonic frequencies, which is by far the dominant part of the computational effort: on the coronene, that calculation requires of the order of two hundred evaluations of the gradient.

A detailed comparison of B3LYP‐3C with literature B3LYP results at various basis set levels reveals that the composite corrections (dispersion, gCP, and short‐range terms) have negligible impact on first ionization energies but become increasingly significant for higher charge states. The systematic underestimation of ionization energies by B3LYP‐based methods is instead dominated by the delocalization error inherent to standard hybrid functionals. Range‐separated functionals such as *ω*B97X‐3C effectively mitigate this error, reproducing the accuracy of M06‐2X calculations performed with substantially larger basis sets.

Overall, our findings show that modern semiempirical and tight‐binding methods, particularly DFTB3, are well suited for studying large PAHs and multiply charged species, enabling the investigation of systems beyond the reach of conventional ab initio methods. For applications requiring higher accuracy on the first ionization energy, *ω*B97X‐3C provides an excellent alternative at moderate computational cost. These results provide practical guidance for selecting computational approaches in astrochemical and molecular astrophysics studies, where PAH size and charge‐state diversity demand both efficiency and reliability.

## Conflicts of Interest

The authors declare no conflicts of interest.

## Supporting information

Supplementary Material

## Data Availability

The optimized geometries of the eight molecules in their neutral, cationic, and dicationic states for the 13 methods benchmarked here, the corresponding total and zero‐point energies, and the scripts that regenerate every table of this work are openly available at https://github.com/ajgiuliani/PAH‐Ionization‐Energies‐Benchmark.
