# Supplementary material for: Ionization Energies of Polycyclic Aromatic Hydrocarbons: Benchmarking Semiempirical, Tight Binding, and Composite Methods
Source: Chemphyschem. 2026 Nov 12;27(21):e70622. doi: 10.1002/cphc.70622 (PMC13760075; doi:10.1002/cphc.70622)
Supplement: Supplementary file 1 [file CPHC-27-e70622-s001.pdf]

Supporting information to Ionization Energies of Polycyclic Aromatic Hydrocarbons: Benchmarking Semi Empirical, Tight Binding and Composite Methods

Alexandre Giuliani
Synchrotron SOLEIL, France
Transform Department, UAR1008, INRA, France
alexandre.giuliani@synchrotron-soleil.fr

Helgi Rafn Hrodmarsson
Laboratoire LISA UMR7583 CNRS, France

1 Description of the supporting information

This document contains the raw energies calculated for all approximate and composite methods (Table S1), the vertical and adiabatic ionization energies with and without zero-point energy corrections (Table S2), the statistical metrics (Table S3), the zero-point energies (Table S4), and the spin contamination of the HF-3C radical cations (Table S5), the dependence of the first ionization energy error on molecular topology (Table S6), the resolution of the statistical metrics (Tables S7 and S8), and the computational cost of the methods as a function of the size of the molecule (Figure S1).

Table S1: Energies (in eV) of the neutral, cation, dication at the equilibrium geometry of the neutral ($R_{Neutral}$), cation (R_{Cation}) and dication ($R_{Dication}$), zero point energy (ZPE) of the neutral, cation and dication.

Molecule	Method	$E_{Neutral}$	E_{Cation}		$E_{Dication}$			ZPE		
		$R_{Neutral}$	R_{Cation}	$R_{Neutral}$	$R_{Dication}$	R_{Cation}	$R_{Neutral}$	Neutral	Cation	Dication
naphthalene	DFTB2	-550.2	-541.7	-541.6	-528.1	-528.0	-527.8	3.994	3.952	3.874
naphthalene	DFTB3	-547.2	-538.8	-538.7	-525.2	-525.1	-524.9	3.927	3.881	3.804
naphthalene	IPEA1	-777.7	-764.6	-764.6	-747.1	-747.0	-746.8	4.421	4.359	4.255
naphthalene	PM6	1.7	10.0	10.2	23.3	23.5	24.1	3.775	3.656	3.578
naphthalene	PM7	1.7	10.1	10.3	23.5	23.6	24.1	3.868	3.759	3.668
naphthalene	RM1	1.6	9.6	9.8	22.7	22.9	23.4	3.985	3.893	3.838
naphthalene	g-xTB	-10500.1	-10492.2	-10492.1	-10478.5	-10478.5	-10478.5	–	–	–
naphthalene	HF-3C	-10364.1	-10356.9	-10356.5	-10343.7	-10343.4	-10342.3	4.579	4.205	4.315
naphthalene	B97-3C	-10495.4	-10487.7	-10487.6	-10474.6	-10474.5	-10474.3	3.942	3.935	3.854
naphthalene	R2SCAN-3C	-10498.0	-10490.2	-10490.1	-10476.8	-10476.7	-10476.5	3.991	3.971	3.913
naphthalene	PBEH-3C	-10477.2	-10469.2	-10469.1	-10455.6	-10455.5	-10455.2	4.144	4.124	4.083
naphthalene	B3LYP-3C	-10492.7	-10485.0	-10485.0	-10471.9	-10471.8	-10471.6	4.006	3.996	3.957
naphthalene	WB97X-3C	-1680.8	-1672.6	-1672.5	-1659.1	-1658.9	-1658.5	4.105	4.085	4.049
fluorene	DFTB2	-711.5	-703.3	-703.2	-690.4	-690.3	-690.1	5.121	5.084	5.015
fluorene	DFTB3	-707.5	-699.4	-699.3	-686.6	-686.5	-686.2	5.031	4.989	4.92
fluorene	IPEA1	-1005.8	-993.0	-992.9	-976.1	-976.0	-975.7	5.656	5.596	5.512
fluorene	PM6	2.1	10.0	10.3	22.7	22.9	23.5	4.813	4.729	4.685
fluorene	PM7	2.1	10.3	10.5	23.0	23.3	23.8	4.93	4.837	4.765
fluorene	RM1	2.0	9.8	10.0	22.3	22.5	23.1	5.08	5.006	4.973
fluorene	g-xTB	-13644.0	-13636.1	-13636.0	-13623.4	-13623.0	-13623.0	–	–	–
fluorene	HF-3C	-13467.5	-13461.0	-13460.5	-13447.9	-13447.6	-13446.5	5.851	5.627	5.621
fluorene	B97-3C	-13637.6	-13630.2	-13630.0	-13617.9	-13617.8	-13617.4	5.022	5.036	5.001
fluorene	R2SCAN-3C	-13641.0	-13633.4	-13633.3	-13620.9	-13620.8	-13620.4	5.085	5.073	5.036
fluorene	PBEH-3C	-13614.2	-13606.4	-13606.2	-13593.6	-13593.4	-13593.0	5.281	5.267	5.240
fluorene	B3LYP-3C	-13634.1	-13626.7	-13626.6	-13614.4	-13614.3	-13613.9	5.105	5.097	5.071
fluorene	WB97X-3C	-2178.7	-2170.8	-2170.6	-2158.0	-2157.8	-2157.3	5.243	5.233	5.216
anthracene	DFTB2	-758.2	-750.5	-750.4	-738.3	-738.2	-738.1	5.273	5.254	5.213
anthracene	DFTB3	-753.9	-746.2	-746.1	-734.0	-734.0	-733.9	5.178	5.157	5.118
anthracene	IPEA1	-1070.7	-1058.5	-1058.5	-1042.4	-1042.3	-1042.2	5.812	5.776	5.721
anthracene	PM6	2.6	10.3	10.5	22.5	22.7	23.1	4.989	4.868	4.8
anthracene	PM7	2.6	10.4	10.6	22.6	22.7	23.2	5.114	5.016	4.945
anthracene	RM1	2.4	9.8	10.0	21.8	21.9	22.4	5.272	5.183	5.148
anthracene	g-xTB	-14680.7	-14673.5	-14673.5	-14660.7	-14660.7	-14660.7	–	–	–
anthracene	HF-3C	-14491.0	-14484.7	-14484.3	-14472.9	-14472.7	-14471.7	6.028	5.690	5.793
anthracene	B97-3C	-14674.1	-14667.2	-14667.1	-14655.6	-14655.6	-14655.4	5.187	5.201	5.166
anthracene	R2SCAN-3C	-14677.8	-14670.7	-14670.7	-14658.9	-14658.9	-14658.7	5.245	5.245	5.219
anthracene	PBEH-3C	-14648.7	-14641.4	-14641.3	-14629.4	-14629.3	-14629.0	5.455	5.454	5.442
anthracene	B3LYP-3C	-14670.5	-14663.6	-14663.6	-14652.0	-14652.0	-14651.8	5.273	5.283	5.274
anthracene	WB97X-3C	-2333.4	-2326.0	-2325.8	-2313.8	-2313.7	-2313.4	5.404	5.404	5.394

Table S1 continued

Molecule	Method	$E_{Neutral}$	E_{Cation}		$E_{Dication}$			ZPE		
		$R_{Neutral}$	R_{Cation}	$R_{Neutral}$	$R_{Dication}$	R_{Cation}	$R_{Neutral}$	Neutral	Cation	Dication
pyrene	DFTB2	-852.8	-845.0	-845.0	-833.0	-833.0	-832.8	5.66	5.638	5.603
pyrene	DFTB3	-847.5	-839.8	-839.8	-827.9	-827.8	-827.6	5.549	5.525	5.49
pyrene	IPEA1	-1202.9	-1190.6	-1190.6	-1174.6	-1174.5	-1174.4	6.203	6.165	6.115
pyrene	PM6	2.7	10.3	10.5	22.3	22.5	23.0	5.344	5.243	5.216
pyrene	PM7	2.7	10.5	10.6	22.4	22.6	23.1	5.484	5.386	5.333
pyrene	RM1	2.4	9.8	10.0	21.5	21.7	22.2	5.651	5.57	5.553
pyrene	g-xTB	-16755.6	-16748.3	-16748.3	-16735.9	-16735.9	-16735.9	–	–	–
pyrene	HF-3C	-16539.8	-16533.7	-16533.3	-16521.8	-16521.6	-16520.6	6.433	6.192	6.207
pyrene	B97-3C	-16747.7	-16740.8	-16740.7	-16729.3	-16729.3	-16729.1	5.540	5.541	5.498
pyrene	R2SCAN-3C	-16752.1	-16745.0	-16744.9	-16733.3	-16733.3	-16733.1	5.599	5.579	5.542
pyrene	PBEH-3C	-16718.9	-16711.6	-16711.5	-16699.7	-16699.6	-16699.3	5.841	5.832	5.825
pyrene	B3LYP-3C	-16743.9	-16737.0	-16736.9	-16725.6	-16725.5	-16725.3	5.645	5.648	5.643
pyrene	WB97X-3C	-2643.9	-2636.5	-2636.3	-2624.5	-2624.4	-2624.0	5.779	5.770	5.766
tetracene	DFTB2	-966.2	-959.0	-958.9	-947.8	-947.7	-947.6	6.548	6.542	6.518
tetracene	DFTB3	-960.4	-953.3	-953.2	-942.1	-942.1	-942.0	6.424	6.417	6.394
tetracene	IPEA1	-1363.8	-1352.1	-1352.0	-1336.8	-1336.8	-1336.7	7.2	7.177	7.14
tetracene	PM6	3.5	10.8	11.0	22.3	22.4	22.9	6.201	6.065	6.017
tetracene	PM7	3.6	11.0	11.1	22.4	22.5	22.9	6.356	6.261	6.199
tetracene	RM1	3.3	10.3	10.5	21.5	21.6	22.0	6.556	6.38	6.435
tetracene	g-xTB	-18861.3	-18854.6	-18854.5	-18843.1	-18843.0	-18843.0	–	–	–
tetracene	HF-3C	-18617.8	-18612.1	-18611.7	-18601.3	-18601.1	-18600.3	7.473	7.140	7.252
tetracene	B97-3C	-18852.7	-18846.3	-18846.3	-18835.8	-18835.8	-18835.7	6.429	6.454	6.444
tetracene	R2SCAN-3C	-18857.5	-18851.0	-18850.9	-18840.3	-18840.2	-18840.1	6.495	6.505	6.498
tetracene	PBEH-3C	-18820.0	-18813.3	-18813.2	-18802.3	-18802.2	-18802.0	6.762	6.771	6.777
tetracene	B3LYP-3C	-18848.2	-18841.9	-18841.8	-18831.3	-18831.3	-18831.1	6.536	6.557	6.566
tetracene	WB97X-3C	-2985.9	-2979.0	-2978.8	-2967.8	-2967.7	-2967.4	6.700	6.709	6.715
coronene	DFTB2	-1250.0	-1242.4	-1242.4	-1231.2	-1231.1	-1231.1	7.705	7.54	7.646
coronene	DFTB3	-1241.5	-1234.0	-1234.0	-1222.8	-1222.7	-1222.7	7.538	7.384	7.473
coronene	IPEA1	-1760.4	-1748.2	-1748.2	-1732.7	-1732.7	-1732.7	8.371	8.23	8.282
coronene	PM6	3.6	11.2	11.4	22.7	23.0	23.1	7.279	7.018	7.026
coronene	PM7	3.7	11.5	11.7	23.0	23.2	23.3	7.474	7.298	7.197
coronene	RM1	3.1	10.5	10.7	21.7	21.9	22.0	7.706	7.52	7.481
coronene	g-xTB	-25086.1	-25078.8	-25078.8	-25067.8	-25067.8	-25067.7	–	–	–
coronene	HF-3C	-24764.3	-24758.3	-24757.9	-24746.6	-24746.4	-24746.2	8.694	8.539	8.954
coronene	B97-3C	-25073.8	-25067.0	-25066.9	-25056.4	-25056.4	-25056.4	7.514	7.495	7.566
coronene	R2SCAN-3C	-25080.6	-25073.6	-25073.6	-25062.9	-25062.8	-25062.8	7.603	7.563	7.611
coronene	PBEH-3C	-25030.9	-25023.6	-25023.5	-25012.4	-25012.3	-25012.3	7.905	7.838	7.820
coronene	B3LYP-3C	-25068.7	-25061.9	-25061.8	-25051.2	-25051.2	-25051.2	7.636	7.577	7.614
coronene	WB97X-3C	-3917.7	-3910.2	-3910.1	-3898.8	-3898.7	-3898.7	7.820	7.751	7.703

Table S1 continued

Molecule	Method	$E_{Neutral}$	E_{Cation}		$E_{Dication}$			ZPE		
		$R_{Neutral}$	R_{Cation}	$R_{Neutral}$	$R_{Dication}$	R_{Cation}	$R_{Neutral}$	Neutral	Cation	Dication
ovalene	DFTB2	-1646.9	-1640.0	-1639.9	-1629.7	-1629.6	-1629.6	9.737	9.726	9.705
ovalene	DFTB3	-1635.1	-1628.2	-1628.2	-1618.0	-1618.0	-1617.9	9.512	9.497	9.475
ovalene	IPEA1	-2317.6	-2306.0	-2306.0	-2291.5	-2291.4	-2291.4	10.525	10.497	10.462
ovalene	PM6	4.9	12.0	12.2	22.7	22.8	23.2	9.188	9.034	9.046
ovalene	PM7	5.1	12.3	12.4	22.9	23.0	23.4	9.444	9.336	9.293
ovalene	RM1	4.2	10.9	11.1	21.4	21.5	21.9	9.738	9.596	9.632
ovalene	g-xTB	-33416.0	-33409.4	-33409.4	-33398.8	-33398.8	-33398.8	–	–	–
ovalene	HF-3C	-32988.3	-32983.4	-32982.9	-32972.8	-32972.6	-32972.0	10.935	10.584	10.725
ovalene	B97-3C	-33399.5	-33393.3	-33393.3	-33383.7	-33383.6	-33383.5	9.453	9.513	9.550
ovalene	R2SCAN-3C	-33408.8	-33402.4	-33402.4	-33392.5	-33392.5	-33392.4	9.559	9.594	9.621
ovalene	PBEH-3C	-33342.5	-33335.9	-33335.8	-33325.6	-33325.6	-33325.4	9.962	9.956	9.958
ovalene	B3LYP-3C	-33393.1	-33386.9	-33386.9	-33377.2	-33377.2	-33377.0	9.618	9.628	9.632
ovalene	WB97X-3C	-5191.1	-5184.3	-5184.1	-5173.9	-5173.8	-5173.5	9.841	9.836	9.845
dicoronylene	DFTB2	-2460.5	-2453.9	-2453.8	-2444.5	-2444.5	-2444.4	14.352	14.346	14.333
dicoronylene	DFTB3	-2442.6	-2436.0	-2436.0	-2426.8	-2426.8	-2426.6	14.01	13.999	13.982
dicoronylene	IPEA1	-3461.7	-3450.4	-3450.3	-3436.7	-3436.6	-3436.5	15.49	15.463	15.435
dicoronylene	PM6	7.2	13.9	14.2	24.1	24.3	24.9	13.546	13.298	13.434
dicoronylene	PM7	7.5	14.6	14.7	24.5	24.7	25.1	13.919	13.749	13.781
dicoronylene	RM1	6.1	12.5	12.8	22.5	22.7	23.2	14.366	14.128	14.276
dicoronylene	g-xTB	-50108.1	-50101.7	-50101.7	-50091.8	-50091.8	-50091.8	–	–	–
dicoronylene	HF-3C	-49467.3	-49465.3	-49464.3	-49452.8	-49451.9	-49451.7	16.103	15.356	15.915
dicoronylene	B97-3C	-50083.1	-50077.2	-50077.2	-50068.6	-50068.5	-50068.4	13.878	13.955	14.007
dicoronylene	R2SCAN-3C	-50097.1	-50091.0	-50091.0	-50082.2	-50082.1	-50082.0	14.011	14.060	14.097
dicoronylene	PBEH-3C	-49997.7	-49991.3	-49991.2	-49982.0	-49981.9	-49981.7	14.651	14.647	14.662
dicoronylene	B3LYP-3C	-50073.7	-50067.8	-50067.7	-50059.0	-50059.0	-50058.8	14.154	14.169	14.184
dicoronylene	WB97X-3C	-7770.3	-7763.7	-7763.5	-7754.2	-7754.1	-7753.7	14.484	14.480	14.509

Table S2: First, second and double ionization energies (in eV) of naphthalene, fluorene, anthracene, pyrene, tetracene, coronene and dicoronylene. Adiabatic energies (aIE) are given with and without zero point energy correction. Vertical ionization energies are labelled as vIE.

Molecule	Method	aIE1 + Δ ZPE	aIE	vIE	aIE2 + Δ ZPE	aIE2	vIE2	aIEd + Δ ZPE	aIEd	vIEd
naphthalene	DFTB2	8.48	8.52	8.59	13.54	13.61	13.68	22.02	22.14	22.4
naphthalene	DFTB3	8.41	8.46	8.52	13.51	13.59	13.66	21.92	22.05	22.31
naphthalene	IPEA1	8.12	8.18	8.25	12.58	12.68	12.75	20.7	20.87	21.14
naphthalene	PM6	8.15	8.27	8.48	13.25	13.33	13.49	21.4	21.6	22.33
naphthalene	PM7	8.28	8.39	8.58	13.27	13.36	13.51	21.55	21.75	22.42
naphthalene	RM1	7.88	7.97	8.18	13.06	13.12	13.26	20.95	21.09	21.78
naphthalene	g-xTB	7.96	7.96	8.03	13.63	13.63	13.63	21.58	21.58	21.65
naphthalene	HF-3C	6.79	7.16	7.59	13.36	13.25	13.54	20.15	20.41	21.83
naphthalene	B97-3C	7.68	7.69	7.76	13.03	13.11	13.19	20.71	20.79	21.1
naphthalene	R2SCAN-3C	7.8	7.82	7.9	13.29	13.35	13.43	21.08	21.16	21.49
naphthalene	PBEH-3C	8	8.02	8.14	13.53	13.57	13.68	21.53	21.59	22.05
naphthalene	B3LYP-3C	7.63	7.64	7.73	13.07	13.11	13.2	20.69	20.74	21.11
naphthalene	WB97X-3C	8.16	8.17	8.31	13.51	13.54	13.68	21.66	21.72	22.26
fluorene	DFTB2	8.18	8.22	8.31	12.77	12.84	12.94	20.95	21.06	21.43
fluorene	DFTB3	8.11	8.15	8.24	12.72	12.79	12.89	20.83	20.94	21.32
fluorene	IPEA1	7.95	8.01	8.11	11.93	12.01	12.11	19.88	20.02	20.43
fluorene	PM6	7.89	7.97	8.19	12.58	12.62	12.85	20.47	20.6	21.45
fluorene	PM7	8.09	8.18	8.38	12.66	12.73	12.95	20.75	20.92	21.71
fluorene	RM1	7.72	7.8	7.99	12.53	12.57	12.76	20.26	20.36	21.12
fluorene	g-xTB	7.91	7.91	7.98	12.67	12.67	13.02	20.57	20.57	20.98
fluorene	HF-3C	6.31	6.53	6.99	13.05	13.06	13.43	19.36	19.59	21.03
fluorene	B97-3C	7.44	7.43	7.54	12.24	12.28	12.39	19.68	19.7	20.15
fluorene	R2SCAN-3C	7.55	7.56	7.68	12.49	12.52	12.64	20.03	20.08	20.56
fluorene	PBEH-3C	7.78	7.79	7.95	12.77	12.8	12.95	20.55	20.59	21.22
fluorene	B3LYP-3C	7.39	7.39	7.52	12.27	12.3	12.43	19.66	19.69	20.21
fluorene	WB97X-3C	7.94	7.95	8.13	12.76	12.77	12.96	20.69	20.72	21.46
anthracene	DFTB2	7.73	7.75	7.79	12.15	12.19	12.23	19.88	19.94	20.11
anthracene	DFTB3	7.66	7.69	7.73	12.1	12.14	12.18	19.76	19.82	20.0
anthracene	IPEA1	7.36	7.4	7.45	11.25	11.3	11.35	18.61	18.7	18.88
anthracene	PM6	7.6	7.72	7.89	12.14	12.2	12.35	19.74	19.93	20.54
anthracene	PM7	7.69	7.79	7.94	12.11	12.18	12.32	19.81	19.98	20.53
anthracene	RM1	7.3	7.39	7.55	11.91	11.94	12.07	19.21	19.33	19.91
anthracene	g-xTB	7.22	7.22	7.29	12.78	12.78	12.78	19.99	19.99	20.06
anthracene	HF-3C	5.97	6.31	6.67	11.88	11.77	12.02	17.85	18.08	19.25
anthracene	B97-3C	6.91	6.89	6.95	11.52	11.56	11.61	18.43	18.45	18.65
anthracene	R2SCAN-3C	7.02	7.02	7.08	11.76	11.78	11.83	18.78	18.81	19.02
anthracene	PBEH-3C	7.24	7.24	7.33	12.05	12.06	12.14	19.28	19.3	19.64
anthracene	B3LYP-3C	6.86	6.85	6.92	11.57	11.58	11.64	18.44	18.44	18.69
anthracene	WB97X-3C	7.41	7.41	7.53	12.11	12.12	12.22	19.52	19.53	19.97

Table S2 continued

Molecule	Method	aIE1 + Δ ZPE	aIE	vIE	aIE2 + Δ ZPE	aIE2	vIE2	aIEd + Δ ZPE	aIEd	vIEd
pyrene	DFTB2	7.72	7.74	7.79	11.99	12.02	12.08	19.71	19.76	19.97
pyrene	DFTB3	7.66	7.68	7.73	11.94	11.97	12.03	19.59	19.65	19.86
pyrene	IPEA1	7.39	7.43	7.48	11.14	11.19	11.25	18.53	18.62	18.84
pyrene	PM6	7.57	7.67	7.85	11.95	11.98	12.15	19.52	19.65	20.34
pyrene	PM7	7.66	7.76	7.92	11.9	11.95	12.11	19.56	19.71	20.34
pyrene	RM1	7.27	7.35	7.53	11.73	11.75	11.89	19.0	19.1	19.73
pyrene	g-xTB	7.23	7.23	7.33	12.4	12.4	12.45	19.63	19.63	19.71
pyrene	HF-3C	5.82	6.06	6.47	11.88	11.86	12.11	17.7	17.92	19.13
pyrene	B97-3C	6.95	6.95	7.01	11.42	11.46	11.52	18.37	18.41	18.64
pyrene	R2SCAN-3C	7.06	7.08	7.15	11.66	11.7	11.76	18.72	18.78	19.02
pyrene	PBEH-3C	7.28	7.29	7.39	11.95	11.96	12.05	19.23	19.25	19.64
pyrene	B3LYP-3C	6.91	6.9	6.98	11.47	11.47	11.55	18.38	18.38	18.67
pyrene	WB97X-3C	7.45	7.46	7.6	11.98	11.98	12.1	19.43	19.44	19.96
tetracene	DFTB2	7.23	7.23	7.26	11.2	11.22	11.25	18.43	18.46	18.57
tetracene	DFTB3	7.16	7.17	7.2	11.13	11.15	11.18	18.29	18.32	18.44
tetracene	IPEA1	6.86	6.88	6.92	10.35	10.39	10.42	17.21	17.27	17.4
tetracene	PM6	7.2	7.34	7.5	11.43	11.48	11.59	18.63	18.82	19.37
tetracene	PM7	7.29	7.38	7.52	11.35	11.42	11.53	18.64	18.8	19.29
tetracene	RM1	6.81	6.98	7.14	11.23	11.18	11.29	18.04	18.16	18.68
tetracene	g-xTB	6.71	6.71	6.78	11.49	11.49	11.59	18.2	18.2	18.33
tetracene	HF-3C	5.35	5.68	6.04	10.9	10.78	10.99	16.25	16.47	17.5
tetracene	B97-3C	6.39	6.36	6.4	10.5	10.51	10.55	16.89	16.87	17.02
tetracene	R2SCAN-3C	6.5	6.49	6.54	10.73	10.73	10.77	17.23	17.22	17.38
tetracene	PBEH-3C	6.71	6.7	6.78	11.04	11.04	11.1	17.75	17.74	18.01
tetracene	B3LYP-3C	6.35	6.33	6.38	10.56	10.55	10.59	16.91	16.88	17.07
tetracene	WB97X-3C	6.9	6.89	7.01	11.16	11.15	11.24	18.06	18.04	18.44
coronene	DFTB2	7.42	7.58	7.6	11.39	11.29	11.31	18.81	18.87	18.92
coronene	DFTB3	7.35	7.51	7.52	11.3	11.22	11.24	18.66	18.72	18.77
coronene	IPEA1	7.24	7.38	7.39	10.64	10.59	10.6	17.88	17.97	18.02
coronene	PM6	7.39	7.65	7.83	11.46	11.45	11.71	18.85	19.11	19.47
coronene	PM7	7.58	7.76	7.91	11.38	11.48	11.71	18.97	19.24	19.56
coronene	RM1	7.2	7.39	7.56	11.16	11.2	11.44	18.36	18.59	18.94
coronene	g-xTB	7.25	7.25	7.31	11.04	11.04	11.04	18.29	18.29	18.35
coronene	HF-3C	5.87	6.03	6.42	12.08	11.66	11.92	17.95	17.69	18.08
coronene	B97-3C	6.82	6.84	6.89	10.62	10.55	10.6	17.44	17.39	17.44
coronene	R2SCAN-3C	6.97	7.01	7.06	10.78	10.73	10.79	17.75	17.74	17.8
coronene	PBEH-3C	7.23	7.3	7.39	11.15	11.17	11.26	18.39	18.47	18.57
coronene	B3LYP-3C	6.79	6.84	6.91	10.66	10.62	10.69	17.44	17.46	17.54
coronene	WB97X-3C	7.43	7.5	7.62	11.33	11.38	11.54	18.77	18.88	19.05

Table S2 continued

Molecule	Method	aIE1 + Δ ZPE	aIE	vIE	aIE2 + Δ ZPE	aIE2	vIE2	aIEd + Δ ZPE	aIEd	vIEd
ovalene	DFTB2	6.95	6.96	6.99	10.28	10.3	10.32	17.23	17.26	17.36
ovalene	DFTB3	6.87	6.89	6.92	10.2	10.22	10.25	17.08	17.11	17.22
ovalene	IPEA1	6.72	6.75	6.78	9.65	9.68	9.71	16.37	16.43	16.54
ovalene	PM6	6.95	7.1	7.29	10.71	10.7	10.79	17.66	17.8	18.32
ovalene	PM7	7.09	7.2	7.33	10.56	10.6	10.7	17.65	17.8	18.26
ovalene	RM1	6.63	6.78	6.95	10.5	10.47	10.54	17.14	17.24	17.71
ovalene	g-xTB	6.61	6.61	6.64	10.58	10.58	10.58	17.19	17.19	17.19
ovalene	HF-3C	4.61	4.96	5.42	10.73	10.59	10.74	15.33	15.54	16.38
ovalene	B97-3C	6.27	6.21	6.25	9.71	9.67	9.69	15.98	15.88	16
ovalene	R2SCAN-3C	6.4	6.36	6.4	9.92	9.9	9.92	16.32	16.26	16.39
ovalene	PBEH-3C	6.62	6.62	6.69	10.24	10.24	10.29	16.86	16.86	17.1
ovalene	B3LYP-3C	6.21	6.2	6.25	9.72	9.72	9.75	15.93	15.92	16.09
ovalene	WB97X-3C	6.81	6.81	6.92	10.36	10.35	10.43	17.16	17.16	17.53
dicoronylene	DFTB2	6.61	6.62	6.65	9.32	9.33	9.37	15.94	15.95	16.09
dicoronylene	DFTB3	6.53	6.54	6.58	9.23	9.25	9.28	15.76	15.79	15.93
dicoronylene	IPEA1	6.45	6.48	6.52	8.84	8.87	8.91	15.29	15.35	15.5
dicoronylene	PM6	6.47	6.72	6.98	10.28	10.14	10.39	16.74	16.86	17.65
dicoronylene	PM7	6.86	7.03	7.2	9.97	9.93	10.11	16.82	16.96	17.6
dicoronylene	RM1	6.13	6.37	6.63	10.17	10.02	10.23	16.3	16.39	17.09
dicoronylene	g-xTB	6.33	6.33	6.37	9.96	9.96	9.96	16.3	16.3	16.3
dicoronylene	HF-3C	1.21	1.96	2.98	13.12	12.56	13.43	14.34	14.52	15.59
dicoronylene	B97-3C	5.96	5.88	5.92	8.7	8.65	8.69	14.66	14.54	14.69
dicoronylene	R2SCAN-3C	6.11	6.06	6.1	8.9	8.87	8.91	15.01	14.93	15.1
dicoronylene	PBEH-3C	6.39	6.39	6.48	9.28	9.26	9.34	15.67	15.66	15.98
dicoronylene	B3LYP-3C	5.93	5.92	5.97	8.73	8.72	8.77	14.66	14.63	14.86
dicoronylene	WB97X-3C	6.64	6.64	6.77	9.46	9.43	9.58	16.1	16.07	16.61

2 Statistical metrics

The statistical metrics are defined as follows:

- Mean signed error: $\text{MSE} = \frac{1}{n}\sum_{i=1}^{n}(x_i - e_i)$
- Mean absolute error: $\text{MAE} = \frac{1}{n}\sum_{i=1}^{n}|x_i - e_i|$
- Root mean square error: $\text{RMSE} = \sqrt{\frac{1}{n}\sum_{i=1}^{n}(x_i - e_i)^2}$
- Standard deviation of the errors: $\sigma = \sqrt{\frac{1}{n-1}\sum_{i=1}^{n}\left[(x_i - e_i) - \text{MSE}\right]^2}$

where x_i and e_i represent the calculated and experimental values, respectively. Whereas the MAE and the RMSE measure how close a method stands on average, σ measures the scatter which remains once the systematic offset has been removed, that is the uniformity of the behaviour of a method along the series. A method may therefore combine a large MSE with a small σ, which indicates a nearly constant offset rather than an erratic behaviour.

Table S3: Statistical metrics obtained from the comparison of the adiabatic ionization energy calculations with experimental data. σ is the standard deviation of the errors.

Methods	MSE	MAE	RMSE	σ
IE1 with ZPE				
DFTB2	0.26	0.26	0.27	0.07
DFTB3	0.19	0.19	0.20	0.07
IPEA1	-0.04	0.05	0.06	0.05
PM6	0.12	0.13	0.15	0.10
PM7	0.26	0.26	0.27	0.08
RM1	-0.15	0.15	0.16	0.06
g-xTB	-0.14	0.14	0.17	0.10
HF-3C	-1.60	1.60	1.61	0.25
B97-3C	-0.49	0.49	0.49	0.05
R2SCAN-3C	-0.37	0.37	0.37	0.06
PBEH-3C	-0.15	0.15	0.16	0.07
B3LYP-3C	-0.54	0.54	0.54	0.05
ωB97X-3C	0.03	0.06	0.07	0.07
IE2 with ZPE				
DFTB2	0.33	0.33	0.34	0.09
DFTB3	0.26	0.26	0.27	0.10
IPEA1	-0.41	0.41	0.43	0.13
PM6	0.60	0.60	0.67	0.32
PM7	0.49	0.49	0.53	0.22
RM1	0.39	0.39	0.51	0.37
g-xTB	0.65	0.65	0.72	0.33
HF-3C	1.04	1.04	1.73	1.52
B97-3C	-0.31	0.31	0.32	0.08
R2SCAN-3C	-0.10	0.12	0.13	0.10
PBEH-3C	0.23	0.23	0.24	0.07
B3LYP-3C	-0.27	0.27	0.28	0.09
ωB97X-3C	0.34	0.34	0.34	0.06
IE double with ZPE				
DFTB2	0.05	0.23	0.27	0.30
DFTB3	-0.07	0.27	0.28	0.30
IPEA1	-1.12	1.12	1.16	0.34
PM6	-0.15	0.27	0.32	0.31
PM7	-0.04	0.22	0.27	0.29
RM1	-0.61	0.61	0.66	0.27
g-xTB	-0.21	0.23	0.27	0.19
HF-3C	-1.71	1.71	1.82	0.68
B97-3C	-1.33	1.33	1.37	0.36
R2SCAN-3C	-0.98	0.98	1.04	0.36
PBEH-3C	-0.46	0.47	0.57	0.36
B3LYP-3C	-1.33	1.33	1.37	0.35
ωB97X-3C	-0.23	0.37	0.40	0.36

Table S4: Zero-point energies (in eV) of the neutral species and ΔZPE = ZPE(cation) − ZPE(neutral) correction to the first adiabatic ionization energy. The xTB-GFN2 values are from the COMPAS database [1, 2].

	Naphthalene	Fluorene	Anthracene	Pyrene	Tetracene	Coronene	Ovalene	Dicoronylene
ZPE of the neutral species								
xTB-GFN2 [1, 2]	3.887		5.111	5.458	6.331	7.370	9.269	
DFTB2	3.994	5.121	5.273	5.660	6.548	7.705	9.737	14.352
DFTB3	3.927	5.031	5.178	5.549	6.424	7.538	9.512	14.010
xTB-IPEA	4.421	5.656	5.812	6.203	7.200	8.371	10.525	15.490
PM6	3.775	4.813	4.989	5.344	6.201	7.279	9.188	13.546
PM7	3.868	4.930	5.114	5.484	6.356	7.474	9.444	13.919
RM1	3.985	5.080	5.272	5.651	6.556	7.706	9.738	14.366
HF-3C	4.579	5.851	6.028	6.433	7.473	8.694	10.935	16.103
B97-3C	3.942	5.022	5.187	5.540	6.429	7.514	9.453	13.878
R2SCAN-3C	3.991	5.085	5.245	5.599	6.495	7.603	9.559	14.011
PBEH-3C	4.144	5.281	5.455	5.841	6.762	7.905	9.962	14.651
B3LYP-3C	4.006	5.105	5.273	5.645	6.536	7.636	9.618	14.154
ωB97X-3C	4.105	5.243	5.404	5.779	6.700	7.820	9.841	14.484
ΔZPE = ZPE(cation) − ZPE(neutral)								
xTB-GFN2 [1, 2]	−0.078		−0.043	−0.044	−0.027	−0.097	−0.028	
DFTB2	−0.042	−0.037	−0.019	−0.022	−0.006	−0.165	−0.011	−0.006
DFTB3	−0.046	−0.042	−0.021	−0.024	−0.008	−0.154	−0.014	−0.011
xTB-IPEA	−0.062	−0.060	−0.036	−0.038	−0.023	−0.141	−0.028	−0.027
PM6	−0.119	−0.084	−0.121	−0.101	−0.136	−0.261	−0.154	−0.248
PM7	−0.109	−0.093	−0.098	−0.098	−0.095	−0.176	−0.108	−0.170
RM1	−0.092	−0.074	−0.089	−0.081	−0.176	−0.186	−0.142	−0.238
HF-3C	−0.375	−0.224	−0.338	−0.241	−0.333	−0.155	−0.351	−0.747
B97-3C	−0.007	0.014	0.014	0.001	0.025	−0.020	0.061	0.077
R2SCAN-3C	−0.020	−0.011	−0.001	−0.020	0.010	−0.040	0.035	0.048
PBEH-3C	−0.020	−0.015	−0.001	−0.009	0.009	−0.067	−0.005	−0.003
B3LYP-3C	−0.010	−0.007	0.010	0.003	0.021	−0.059	0.009	0.014
ωB97X-3C	−0.020	−0.010	−0.000	−0.009	0.009	−0.069	−0.005	−0.005

Table S5: Spin contamination of the HF-3C open-shell species. $\langle \hat{S}^2 \rangle$ is the expectation value returned by ORCA, to be compared with the ideal value of 0.75 for a doublet and of 2.00 for a triplet. $\Delta E = E(\text{ROHF}) - E(\text{UHF})$ is evaluated at the same geometry. Since ROHF is a constrained UHF, ΔE is positive by construction and measures the energy gained by the solution of broken symmetry. R_c and R_n denote the geometries of the cation and of the neutral, R_d that of the dication. The corresponding values obtained for the five density functional based composite methods, with the identical protocol, remain below 0.01 eV.

	At R_c			At R_n		
Molecule	$\langle \hat{S}^2 \rangle$	− ideal	ΔE (eV)	$\langle \hat{S}^2 \rangle$	− ideal	ΔE (eV)
Doublet radical cations						
Naphthalene	0.877	0.127	0.366	0.845	0.095	0.295
Fluorene	1.680	0.930	0.927	1.532	0.782	0.714
Anthracene	0.907	0.157	0.398	0.867	0.117	0.329
Pyrene	1.353	0.603	0.798	1.251	0.501	0.634
Tetracene	0.960	0.210	0.453	0.909	0.159	0.374
Coronene	1.881	1.131	0.967	1.735	0.985	0.822
Ovalene	2.466	1.716	1.298	2.016	1.266	0.910
Dicoronylene	6.859	6.109	4.778	5.874	5.124	3.255
Triplet dication of coronene						
Coronene (R_d)	2.053	0.053	0.156			
Coronene (R_c)	2.089	0.089	0.168			
Coronene (R_n)	2.050	0.050	0.154			

Table S6: Deviation from experiment of the first adiabatic ionization energy (in eV, ZPE-corrected) for the two structural families of the benchmark set: the linear acenes and the pericondensed PAHs. The last two columns give the total drift over each subseries, that is the deviation of the largest member minus that of the smallest one. The error grows steadily along the acene series, whereas it remains essentially constant for the pericondensed species, although the latter span a wider range of carbon numbers.

	Linear acenes			Pericondensed			Drift	
Method	Naphth. C_{10}	Anthr. C_{14}	Tetrac. C_{18}	Pyrene C_{16}	Coron. C_{24}	Ovalene C_{32}	acenes	peri.
DFTB2	0.34	0.29	0.26	0.29	0.13	0.24	−0.08	−0.05
DFTB3	0.27	0.23	0.19	0.23	0.06	0.16	−0.08	−0.06
IPEA1	−0.02	−0.08	−0.11	−0.04	−0.05	0.01	−0.09	0.05
PM6	0.01	0.16	0.23	0.15	0.10	0.24	0.23	0.09
PM7	0.14	0.26	0.32	0.23	0.29	0.38	0.18	0.15
RM1	−0.26	−0.14	−0.16	−0.15	−0.09	−0.08	0.10	0.08
g-xTB	−0.19	−0.22	−0.26	−0.19	−0.04	−0.10	−0.07	0.09
HF-3C	−1.36	−1.47	−1.62	−1.61	−1.42	−2.10	−0.26	−0.50
B97-3C	−0.46	−0.53	−0.58	−0.48	−0.47	−0.44	−0.12	0.04
R2SCAN-3C	−0.35	−0.42	−0.47	−0.37	−0.32	−0.31	−0.12	0.06
PBEH-3C	−0.14	−0.20	−0.26	−0.14	−0.06	−0.09	−0.12	0.05
B3LYP-3C	−0.52	−0.57	−0.62	−0.52	−0.50	−0.50	−0.11	0.02
ωB97X-3C	0.01	−0.03	−0.07	0.03	0.14	0.10	−0.08	0.07

3 Resolution of the statistical metrics

The metrics of Table S3 rest on seven, six and six molecules for the first, second and double ionization energies respectively, and the reference values carry experimental uncertainties u_i that reach 0.2 eV for the double ionization energy (Tables 1 to 3 of the article). Two independent sources of error therefore limit the precision with which the mean absolute error (MAE) of a method is known: the finite size of the reference set and the uncertainty of the reference values. Each has been estimated separately, and the two have then been combined. In every case the MAE is recomputed 2×10^5 times and the half-width of the interval containing 95 % of the recomputed values is reported. The results are summarized in Table S7 and given for each method in Table S8.

Sample size. The n molecules having an experimental value are drawn with replacement among themselves in a classical bootstrap procedure, the calculated and reference values being kept, and the MAE is recomputed on the set obtained. This measures how much the MAE depends on which molecules happen to compose the set. It is the resampling counterpart of the standard error of the MAE, $\mathrm{SE} = s/\sqrt{n}$, where s is the sample standard deviation of the absolute errors $|x_i - e_i|$. We note, however, that with six or seven molecules the bootstrap interval is known to be somewhat too narrow.

Experimental uncertainty. The set of molecules is kept unchanged and each reference value e_i is replaced by a draw from $\mathcal{N}(e_i, u_i^2)$, the calculated values being held fixed. This measures how much the MAE would change if the reference values were measured again within their experimental uncertainty. The quoted uncertainties are taken as one standard deviation. This contribution is small for the first ionization energy and comparable to or larger than the sample-size contribution for the second and double ionization energies.

Both combined. The two sources being independent, their half-widths are added in quadrature, $U = \sqrt{U_{\mathrm{size}}^2 + U_{\mathrm{exp}}^2}$. This combination should be read as an upper estimate.

Two MAE, each known to within the combined half-width U, can be told apart only if they differ by more than about $\sqrt{2}\,U$. With the median values of U (0.05, 0.11 and 0.25 eV, Table S7), differences in MAE below about 0.1, 0.15 and 0.3 eV for the first, second and double ionization energies respectively are therefore not significant, and are not used to rank methods. The calculation may be reproduced with the script `resampling.jl` of the data deposit.

Table S7: Estimated 95 % uncertainty (half-width, in eV) of the mean absolute error, from the finite size of the reference set alone (bootstrap over molecules), from the quoted experimental uncertainties alone (Monte-Carlo resampling of the reference values at fixed calculated values), and from both combined. Values are representative (median over the thirteen methods).

	Sample size	Experimental	Combined
IE1 ($n = 7$)	0.04	0.02	0.05
IE2 ($n = 6$)	0.07	0.09	0.11
IE double ($n = 6$)	0.19	0.15	0.25

Table S8: Mean absolute error (MAE, in eV) of each method and its 95 % uncertainty (half-width, in eV) from the finite size of the reference set alone, from the quoted experimental uncertainties alone, and from both combined.

Methods	MAE	Sample size	Experimental	Combined
IE1 ($n = 7$)				
DFTB2	0.26	0.04	0.02	0.05
DFTB3	0.19	0.04	0.02	0.05
IPEA1	0.05	0.02	0.02	0.03
PM6	0.13	0.06	0.02	0.07
PM7	0.26	0.06	0.02	0.06
RM1	0.15	0.04	0.02	0.05
g-xTB	0.14	0.06	0.02	0.07
HF-3C	1.60	0.17	0.02	0.17
B97-3C	0.49	0.03	0.02	0.04
R2SCAN-3C	0.37	0.04	0.02	0.04
PBEH-3C	0.15	0.05	0.02	0.05
B3LYP-3C	0.54	0.03	0.02	0.04
ωB97X-3C	0.06	0.03	0.02	0.04
IE2 ($n = 6$)				
DFTB2	0.33	0.07	0.09	0.11
DFTB3	0.26	0.07	0.08	0.11
IPEA1	0.41	0.09	0.09	0.13
PM6	0.60	0.23	0.09	0.24
PM7	0.49	0.16	0.09	0.18
RM1	0.39	0.26	0.09	0.27
g-xTB	0.65	0.24	0.09	0.26
HF-3C	1.04	1.04	0.09	1.04
B97-3C	0.31	0.06	0.09	0.11
R2SCAN-3C	0.12	0.05	0.07	0.09
PBEH-3C	0.23	0.05	0.08	0.10
B3LYP-3C	0.27	0.06	0.09	0.11
ωB97X-3C	0.34	0.04	0.09	0.10
IE double ($n = 6$)				
DFTB2	0.23	0.12	0.13	0.18
DFTB3	0.27	0.08	0.14	0.16
IPEA1	1.12	0.25	0.16	0.30
PM6	0.27	0.13	0.14	0.19
PM7	0.22	0.11	0.13	0.17
RM1	0.61	0.19	0.15	0.25
g-xTB	0.23	0.11	0.13	0.17
HF-3C	1.71	0.49	0.16	0.52
B97-3C	1.33	0.26	0.16	0.30
R2SCAN-3C	0.98	0.26	0.16	0.31
PBEH-3C	0.47	0.25	0.14	0.29
B3LYP-3C	1.33	0.25	0.16	0.30
ωB97X-3C	0.37	0.12	0.15	0.19

4 Computational costs

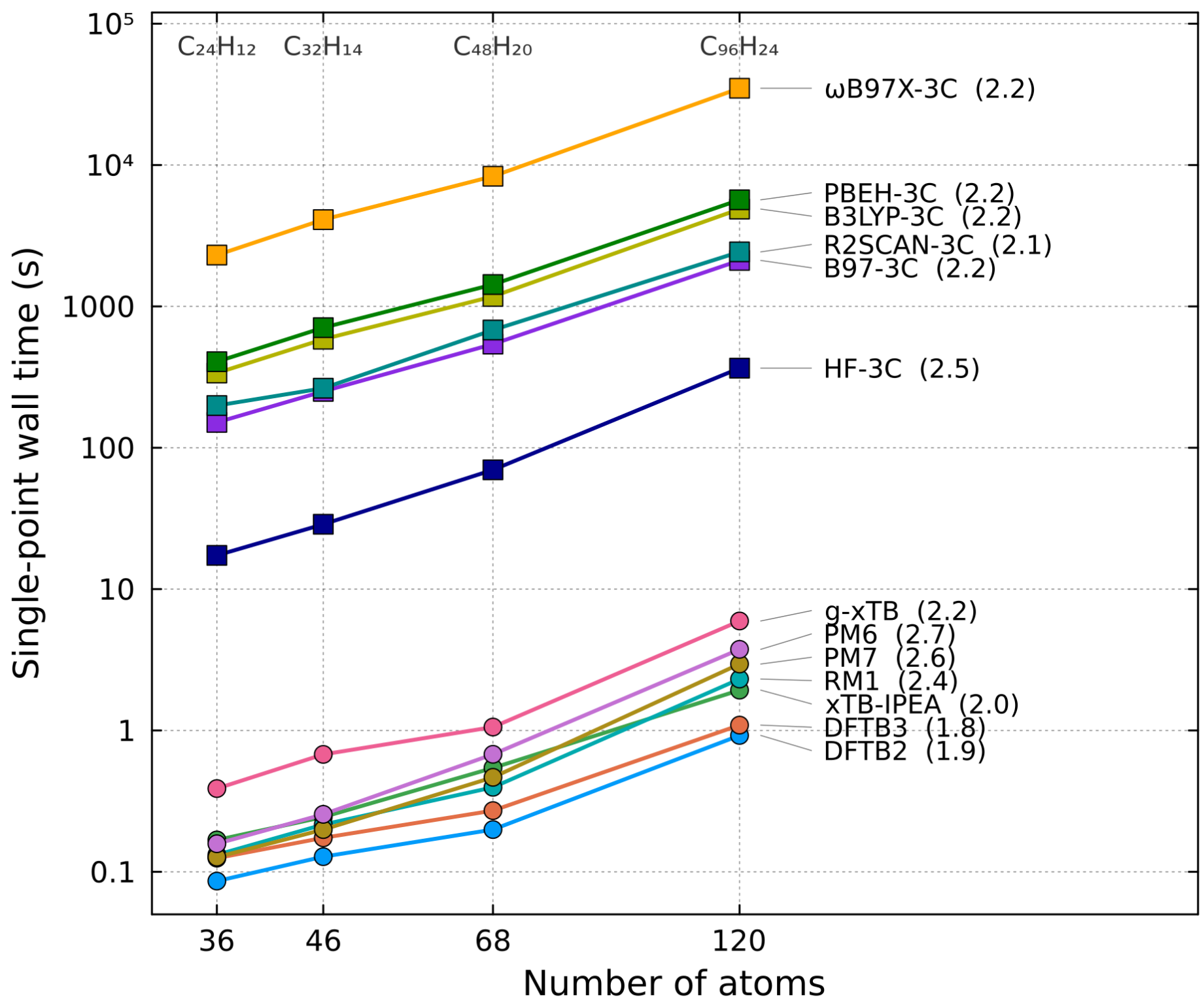

Figure S1: Wall time of a single-point energy evaluation against the number of atoms, on log–log axes, for coronene ($C_{24}H_{12}$), ovalene ($C_{32}H_{14}$), dicoronylene ($C_{48}H_{20}$) and circumcircumcoronene ($C_{96}H_{24}$). Each calculation starts from scratch and runs on a single core of an Intel Xeon E5-1650 v2 processor. Each point is the median of five trials (semi-empirical and tight-binding methods) or three trials (g-xTB and composite methods), except for ωB97X-3C on $C_{96}H_{24}$ (a single trial of 9.7 h). The number in parentheses is the effective exponent α, i.e. the slope of $\ln t$ against $\ln N$ over the four sizes. The individual timings are given in the file `timings_size_scaling.txt` of the data deposit. The times are orders of magnitude: they depend on the machine, on the program and on its settings.

References

[1] A. Wahab, L. Pfuderer, E. Paenurk, R. Gershoni-Poranne, *Journal of Chemical Information and Modeling* **2022**, *62*, 3704.

[2] A. Wahab, R. Gershoni-Poranne, *Physical Chemistry Chemical Physics* **2024**, *26*, 15344.
